# pvsR: An Open Source Interface to Big Data on the American Political Sphere

**DOI:** 10.1371/journal.pone.0130501

**Published:** 2015-07-01

**Authors:** Ulrich Matter, Alois Stutzer

**Affiliations:** University of Basel/Faculty of Business and Economics, Peter-Merian-Weg 6, 4002 Basel, Switzerland; Instituto de Fisica Interdisciplinar y Sistemas Complejos IFISC (CSIC-UIB), SPAIN

## Abstract

Digital data from the political sphere is abundant, omnipresent, and more and more directly accessible through the Internet. Project Vote Smart (PVS) is a prominent example of this big public data and covers various aspects of U.S. politics in astonishing detail. Despite the vast potential of PVS’ data for political science, economics, and sociology, it is hardly used in empirical research. The systematic compilation of semi-structured data can be complicated and time consuming as the data format is not designed for conventional scientific research. This paper presents a new tool that makes the data easily accessible to a broad scientific community. We provide the software called pvsR as an add-on to the R programming environment for statistical computing. This open source interface (OSI) serves as a direct link between a statistical analysis and the large PVS database. The free and open code is expected to substantially reduce the cost of research with PVS’ new big public data in a vast variety of possible applications. We discuss its advantages vis-à-vis traditional methods of data generation as well as already existing interfaces. The validity of the library is documented based on an illustration involving female representation in local politics. In addition, pvsR facilitates the replication of research with PVS data at low costs, including the pre-processing of data. Similar OSIs are recommended for other big public databases.

## Introduction

In recent years, the dawn of the new discipline of ‘computational’ social science has been widely discussed (see, e.g., [1, 2, 3, 4]). This has been brought about by increased computational power and immensely rich sources of digital data covering every-day human activities such as consumer histories from online shopping outlets, records of social interactions based on web platforms such as Facebook and Twitter, and medical histories via the digitization of health insurance processes, as well as tracks of geographic movements recorded by mobile applications on smart phones (these developments and the applications of related research methods are also referred to as eScience and data science). While such data offer exciting potential for quantitative research in diverse fields such as sociology, economics, political science and social psychology, the associated privacy concerns are not to be underestimated (see, e.g., [5, 6, 7]). There is, however, a comparatively smaller but nevertheless important domain of data sources which has received limited attention from the emerging computational social science community so far; i.e., big data on the public and political sphere. These data pose only minor ethical concerns, yet offer major advantages.

A prominent example are the data offered by Project Vote Smart (PVS). PVS provides the public in the United States with detailed information on various political issues at all levels of government via its web platform www.votesmart.org and thereby maintains a detailed online data collection on multiple aspects of U.S. politics. The idea behind PVS’ web platform is to increase transparency of the political process and thereby facilitate voters’ decisions. An important aspect for social scientists is that PVS not only serves as a stage for elected officials but also for candidates running for a public office. Thereby, the central research subjects provide a large amount of data about themselves, including details on their biographical background and political opinions. Separately, large records of these politicians’ voting behavior and other actions in office are collected. Despite the vast potential of PVS’ data for diverse fields in the social sciences, they have hardly been used for scientific studies. The reason is probably that their use is not straightforward. Technically, access is facilitated via an application programming interface (hereafter API).

We understand the term (web) API as it is used in the web development context, i.e., a collection of defined HTTP requests and their respective response messages as documents in Extensible Markup Language (XML) or JavaScript Object Notation (JSON) format for the purpose of exchanging data over the Internet. More specifically, we refer here to APIs based on the representational state transfer (REST) principle (see [8] for a general introduction to RESTful web APIs). The term ‘web service’ is often used as synonym for an API in the web development context and stresses the server-side implementation of APIs. Client-side applications, also referred to as API client libraries, are programs written to either manually or automatically interact with an API (i.e., in the form of sending requests to the API and handling the returned web data). Typical API client libraries in the context of web development facilitate the embedding of web data provided via the API in dynamic websites. API client libraries are often written in the programming languages that are frequently used in web development such as PHP or Java.

The PVS API is predominantly provided for the web and software developers who write mobile applications and dynamic websites which embed PVS data. Due to the primary intended use of the data, its format is not designed for conventional scientific research. The data entries first need to be parsed and formatted in a (table-like) flat representation. The compilation of such semi-structured web data for scholarly analyses can thus be complicated and requires a scientific understanding of the information in the data *as well as* a neat computational background.

In this article, we introduce a software package called pvsR that automates the systematic compilation and transformation of data from PVS via its API for scientific analysis. The sofware is freely available as an R-package for academic/non-profit purposes. It is platform-independent and can be directly installed from the R command line with the command install.packages(’pvsR’) or downloaded from http://CRAN.R-project.org/package=pvsR. See [9] for details on the R-package. This free open source interface is expected to substantially reduce the costs of research using new big public data provided by PVS.

We use the term Open Source Interface (hereafter OSI) to describe the API client libraries that are specifically tailored for social science research. Such libraries work as well-documented add-ons to statistical software packages such as R and offer well-guided high-level access to data from a web service. An OSI substantially facilitates the compilation of data drawn from an API that was not initially designed to provide data for statistical analysis, but was instead designed for integration in dynamic websites and smart phone applications. OSIs thus preprocess, flatten, bind, and link the raw tree-structured web data into convenient table-like formats for statistical analysis. We use the term big data, as defined by Michael Franklin (in [10]: 4), for data that is “expensive to manage” and “hard to get value from”. While the size of such data (in terms of bytes) is a challenge, it is by far not the only one. The data format, for example, is often the biggest obstacle to analysis. In the case of big public data, the initial purpose the data is collected for, usually differs from the purpose of the analysis with this data later on, often requiring a completely different data structure. This is particularly the case for many recent studies in the social sciences that rely on big data collected from programmable web sources such as the Twitter API. There are, of course, other definitions of the term big data, depending on the academic field or the area of application (see, e.g., the expert survey conducted by the Berkeley School of Information: http://datascience.berkeley.edu/what-is-big-data/). In particular, complexity might be added as a substantial attribute to the definition of big data. In the future, new public data sources from government offices might best be qualified as big public data due to this attribute.

The application of pvsR in social science research on U.S. politics offers various advantages over existing data-gathering methods and research practices. First, pvsR provides instant access to highly granular data on all levels of government in the United States in a format that can be directly integrated in a statistical analysis. From the U.S. President to a local council member, data on officials is made easily accessible in the same format and includes detailed biographical information such as gender, professional experience, or religious affiliation. Second, compiling and coding data via pvsR (as well as via any other API-client for PVS) can replace the querying of candidates and officials which is often loaded with a specific survey context. The provision of the raw data to PVS by the individual candidates and officials happens completely independently of any later ‘computational surveying’ by a researcher via pvsR. The raw information is often not forced into predefined categorical answers and is less likely to suffer from response bias than ordinary survey data. Third, the application of pvsR automatically facilitates the replicability of the resulting research. This is a crucial aspect, as there is currently a paradoxical development regarding the transparency of research in the context of social science with big data from the Internet. With the increase in publicly available big data, there are also more studies which base their research on “unique” and “original” datasets. While this development is welcome, the new studies are harder to replicate than empirical analyses based on traditional sources from official statistics. Even if a dataset is made available, there are substantial up-front costs associated with data selection and data cleaning when a dataset has to be reconstructed or extended. In the case of research with PVS data, pvsR substantially reduces the costs of generating datasets for reproduction and replication. As pvsR starts at the data retrieval stage, the pre-processing of data is automatically included when research is reproduced. Simply providing the code of how pvsR was applied in a study is a sufficient documentation of what raw data was used and how it can be accessed for a reproduction or a replication of the study. We illustrate some of the advantages of our software approach compared to traditional methods of data generation based on an application on the representation of women in local politics.

The developed arguments suggest the application of OSIs also in connection with other data bases offering access via an API. It simplifies their use and allows studies to be replicated based on this new data. A look at the emerging empirical literature that exploits publicly available big data on various aspects of society outside the political sphere indicates the potential of OSIs. In particular, popular social media sites such as Twitter, Facebook, and Weibo, as well as Wikipedia are ingcreasingly used as data sources for quantitative analyses covering many aspects of society. Recent research has shown, for example, that stock market moves can be predicted by sentiment analysis of micro-blog messages [11], the analysis of Wikipedia usage patterns [12], as well as the analysis of specific Google search volumes [13]. In [12], Wikipedia usage patterns are analyzed as an approximation of investors’ information gathering to detect early signs of stock market moves. The authors present evidence suggesting that the number of page views of Wikipedia articles related to financial topics increases before stock market falls. In [14], new approaches are presented to measure labor market flows by creating indexes of job loss, job search, and job posting based on data from Twitter. Other studies based on newly available big data aim, for example, at the quantification of an individual’s mood and happiness based on a sentiment analysis of tweets ([15, 16] and [17]) as well as the study of collective human attention on natural disasters via Flickr-tags [18].

The remainder of this paper is organized as follows. In Section 2, we present an overview regarding access to data via APIs. Moreover, we introduce the PVS API as well as the basic idea behind pvsR. In Section 3, we show how to work with pvsR and present a working example. This example documents how data gathering with pvsR supersedes existing data collection practices in political science. In Section 4, we demonstrate pvsR’s advantages in terms of replicability of research by replicating a part of an existing study based on PVS data. Section 5 provides some technical background to pvsR and Section 6 compares its core features to other API client libraries for PVS not particularly developed for social science research and written in other programming languages. The concluding discussion in Section 7 summarizes the advantages of pvsR and OSIs in general, based on a conceptual perspective, and points out what could be done to foster the provision of OSIs to big public data.

## Background

The costs of gathering and distributing information for the public’s political engagement have significantly fallen with higher Internet penetration. Democratic movements now have numerous opportunities to pursue their political goals using the Internet and social media. As a consequence, entries in political blogs and tweets leave electronic traces which can be used to generate, for example, a new kind of data on political opinion formation and behavior [19], on political polarization [20], as well as on political discourse [21] (note that the author of [20] also contributes an OSI-like R package to compile data from the Twitter Streaming API [22].). These data are explicitly meant to be publicly accessible (and the dissemination is approved by the individuals generating it). Moreover, the Internet has given rise to citizens’ groups and non-governmental organizations whose aim it is to make the democratic process more transparent. These bodies gather data, for example, on political candidates, public officials, and campaign finances in order to inform voters.

This development offers researchers who want to investigate the public and political sphere various opportunities to undertake descriptive analyses as well as hypothesis testing. New descriptive insights into the structure of the political system are expected, as computationally intense methods from fields such as network science are applied to vast data sets (see, e.g., [23] who unveils the topology of legislators’ co-sponsership networks based on all 280,000 items of legislation proposed in the U.S. Congress between 1973 and 2004). Regarding hypothesis testing, new light can be shed on existing theories, because more concepts and variables, such as those relating to campaign contributions and political behavior, can be empirically captured and measured.

Data generated by the processes described above are generally published on dynamic websites to inform the public on diverse political issues. The editing and presentation of the data is optimized for individual users interested in a particular issue or person. Hence, the normal user accesses the data through his or her web browser or a similar device with a graphical user interface (e.g., getting information on the candidates in a specific electoral district via PVS’s website). Gathering web data in such a manual way is also still a wide spread practice in the social sciences. Data is retrieved piecewise with a single query or repeated queries through a web browser, after which the data can then be stored in a spreadsheet to build a unique data set.

**Fig 1:**
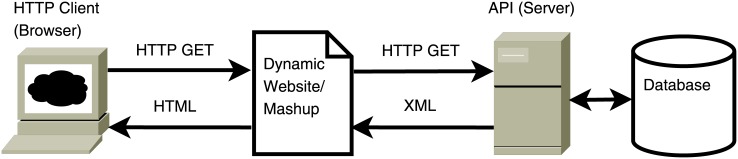
APIs as part of a dynamic website.

However, web data sources can also be accessed increasingly through an API, which enables specific queries to be run programmatically on the data (instead of extracting the information from hundreds of web pages). A prominent example is the initiative PVS (see http://www.votesmart.org.). PVS is a non-profit organization engaged in informing citizens about U.S. politics. The organization is non-partisan and collects and distributes information ranging from local to federal elections and voting on ballot measures. Of particular relevance is the background information that it collects on candidates and elected officials. This involves information about their previous occupations, education, family life, organizational memberships and, importantly, also their voting behavior in key votes at the federal and state level. PVS also asks politicians about their political position, based on a standardized so-called Political Courage Test. Furthermore, information about campaign finances, interest group ratings of politicians and ballot measure descriptions for states with direct legislation is provided. PVS offers its data (subject to an annual registration fee) free of charge and provides a set of tools that allow users to integrate specific items of information in their web pages (see, e.g. [24]). Access is granted via an API that is developed on the REST architectural style. Such APIs usually return data in a hierarchically (or tree-)structured XML or JSON format. Data in such a format can easily be embeded in other websites (i.e., so-called mashups) or implemented in mobile phone applications (in the case of PVS, e.g., AT&T’s 2010 VoterHub Mobile App; see http://voterhub.us/). This is also the main motivation for non-governmental organizations like PVS for opening access to the public through an API. The dissemination of information is part of their mission to increase transparency in the political process. Besides PVS, there are various other NGOs offering access to public data on U.S. politics via an API. For example, the Center for Responsive Politics (CRP) which provides (through their database OpenSecrets.org) detailed information on federal campaign contributions and lobbying activities in the United States (http://www.opensecrets.org/resources/create/apis.php) and MapLight whose Bill Positions API offers access to data on organizations’ interests for legislative bills (http://maplight.org/apis/bill-positions). A good example of a company that offers access to data on the public sphere is the New York Times (NYT). The APIs offered by NYT provide data on various aspects of U.S. federal and state politics, as well as on news and readers’ comments (http://developer.nytimes.com/docs/read/Home). Finally, as an example of an API provided by a public authority, the Parliamentary Services of Switzerland offer access to data on diverse parliamentary activities (http://www.parlament.ch/e/dokumentation/webservices-opendata/pages/default.aspx). Fig 1 illustrates the conventional use of APIs as an integrated component of a dynamic website.

The design of APIs facilitates web and software developers’ access to the data in order to embed the data in their applications. However, its design is not per se suited to systematic scientific data compilation and analysis (an exeption is, of course, the type of APIs that are specifically designed by research institutes for the storage and exchange of data sets between academics). In most cases, the tree-structured data returned from such an API cannot be analyzed directly using standard statistical procedures. Accordingly, another interface is needed to make such data easily accessible to researchers; i.e., an interface that translates the tree-structured data provided by the API to a corresponding flat data representation. This is also the case with the PVS API. To overcome this challange, we provide pvsR as an OSI to the PVS API. Other OSI-like client libraries for other APIs are, e.g., the Sunlight Foundation’s Python library to the Influence Explorer API [25] and the R package WDI [26].

**Fig 2:**
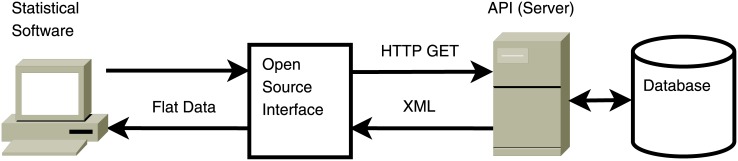
Open source interface to an API.

pvsR automates the data retrieval requests to the PVS API, handles HTTP and API errors as well as malformed XML, reshapes the data format, and stores it or makes it available for econometric analyses. In addition, several high-level functions are provided that combine different API methods and conveniently allow for fetching data on specific data entity. Another interface is thus created which bridges data retrieval and analysis. Fig 2 illustrates this alternative use of APIs to web platforms such as PVS for social science research.

## Compilation of big data on U.S. politics: An introduction to pvsR

The pvsR package is written as an add-on to the R statistical computing environment, guaranteeing platform independence and free availability. It is completely open source (released under the GPL-2 license) and is made available in the CRAN repository. pvsR consists of around 60 well-documented functions. These include, for example, Candidates.getByLastname(), which takes a candidate’s last name as an input variable (or a list of candidates’ last names) and returns a data frame with detailed data on the candidate(s) matching that last name(s). In the Supporting Information (S1 Tutorial) to this article, a tutorial for pvsR is provided. Together with the online documentation in the R repository (http://cran.r-project.org/web/packages/pvsR/pvsR.pdf), this is sufficient so that the PVS API can easily be queried for scientific research. In this section, we first explain how to install and work with the OSI pvsR. We then present in a working example how the relatively simple retrieval and aggregation of high-quality data on U.S. politics from the PVS API via pvsR has the potential to replace common survey practices in the field of political science.

### Installation of pvsR and basic functionality

The easiest way to install pvsR is to use the following command in the R-console (R version ≥ 3.0 needed):

*> install.packages(’pvsR’)*

Alternatively, the package can also be downloaded as source codes or binaries from CRAN (http://CRAN.R-project.org/package=pvsR) and installed from the locally saved file.

Most query functions in the pvsR package take a simple input argument and return the data describing this input variable in a data frame (i.e., a spread sheet-like R-object suitable for statistical analysis). Note that all pvsR functions automatically handle several input arguments (which implies various HTTP requests to the API). We start with a simple example to demonstrate the basic functionality. The function Officials.getByLastname() takes the last name(s) of one (or several) U.S. officials as an input variable and returns a data frame with a row for each official and columns describing his or her profile.

*> library(pvsR)*

*> pvs.key <- “YOUR-PVS-KEY”*

*> ob <- Officials.getByLastname(lastName = list(“Obama”,“Boehner”))*

*> print(ob[,1:6])*

candidateId firstName nickName middleName preferredName lastName

1 9490 Barack Hussein Barack Obama

2 27015 John A. John Boehner

Note that it is mandatory to have a PVS API key in order to access any data (independent of the tool used to access the data). After installing and loading the pvsR package, make sure that your personal PVS API key is saved in a variable called pvs.key as shown in the code above. All query functions will automatically refer to this variable afterwards. See http://votesmart.org/share/api on how to register with Project Vote Smart in order to obtain an API key.

### Instant access to highly granular data: A study of female representation in local U.S. politics

Combining different pvsR functions and basic R functions to retrieve and aggregate information about U.S. politics in order to answer simple empirical questions is straightforward. For example, what is the current fraction of female legislators in the California State Senate? In order to answer this question, we first use Officials.getByOfficeState() to obtain a data frame with all current senators in the California State Senate. The parameter officeId specifies the office from which data should be provided, 9 stands for State Senate (see http://api.votesmart.org/docs/semi-static.html for a complete list of available offices and their respective ID-number). In a second step, we simply take the unique identification numbers (candidateId) of all these senators to query biographical information on each one of them with the function CandidateBio.getBio().

*> ca senators <- Officials.getByOfficeState(stateId = “CA”, officeId = 9)*

*> sen bio <- CandidateBio.getBio(candidateId = ca senators$candidateId)*

*> print(summary(sen_bio$candidate.gender))*

Male Female

26 11

Note the high accuracy of the information aggregated in this manner. At the time this document was generated (February, 2015), the total count of senators according to the method above was 37. Although the California State Senate has 40 seats, 37 was absolutely correct. Three of the seats (districts 07, 21, and 37) were vacant at this point in time [27].

**Fig 3:**
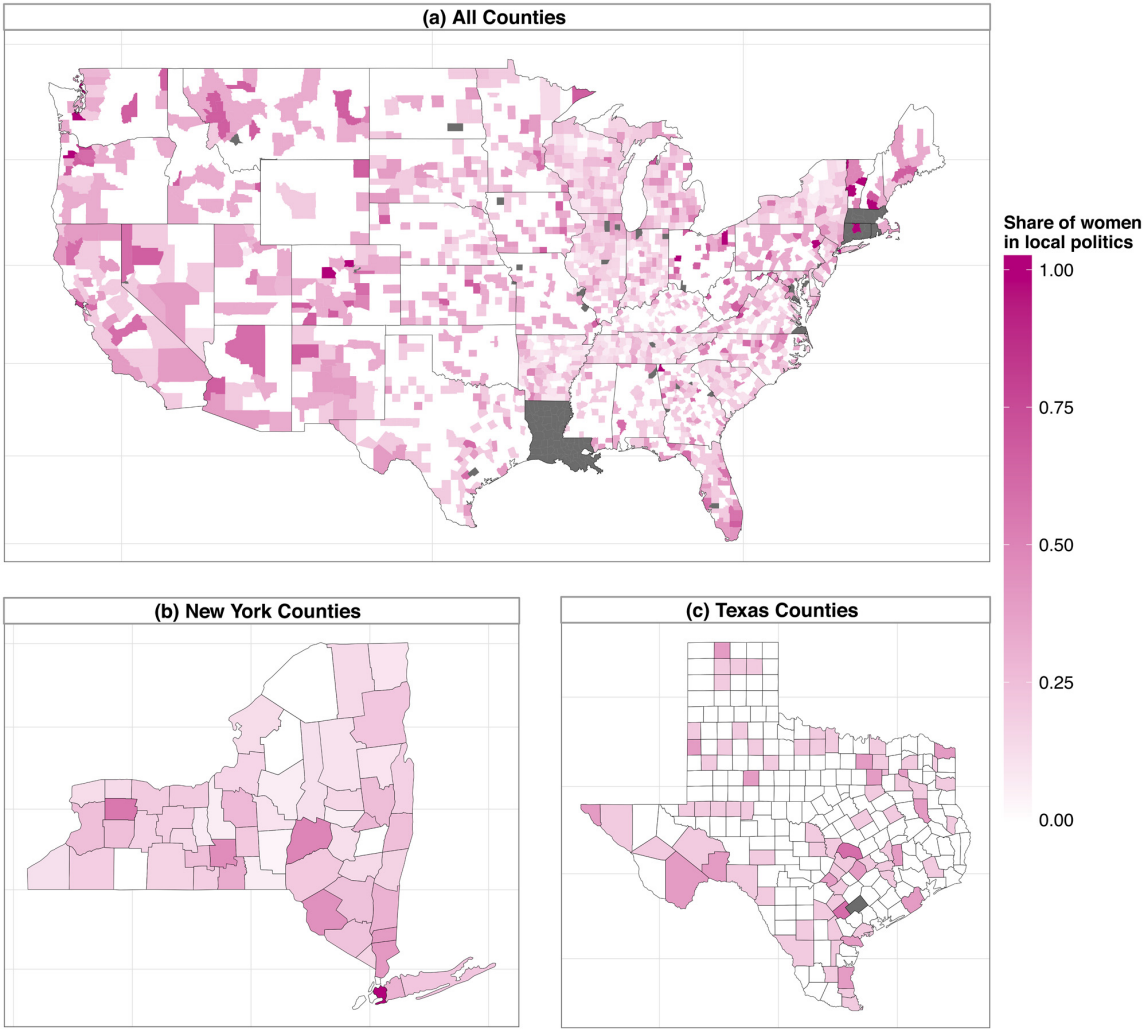
The current share of women in county legislative offices across the United States. *Data sources:* Own compilation based on Project Vote Smart using pvsR.

The study of women in politics (in and outside the United States) is so far primarily focused on the national or the state level. However, the few studies analyzing gender differences in local politics show that politicians’ gender seems to matter a lot for local policy outcomes (see, e.g., [28] and [29]). Studies that focus on local politics in the United States often appear (at least in part) to be shaped by data availability issues by focusing, for example, only on mayors of rather big cities or on a relatively small sample based on a local survey (see, e.g., [30] and [31]). Classical data sources on women in U.S. politics cover data on federal and state offices over many years (see as a reference point the Center for American Women in Politics of the Eagelton Institute of Politics at the Rutgers University). Apart from data on city executives, however, data on local politicians is not instantly available from classical data sources. We show how the simple approach to instantly query data on politicians’ genders via pvsR can easily be extended to a thorough inquiry regarding female representation in county legislative offices across the United States. To the best of our knowledge, this is the first study to unveil the share of women in local U.S. politics to such a highly granular extent. To do so, we first gather the raw PVS data on all county officials with the high-level function getAllLocalOfficials(locality = counties) (see the next section for more details on high-level functions in pvsR.) and query all biographical data on all those officials via CandidateBio.getBio(). As not all biographical profiles reveal the officials’ gender, we match the officials’ first names with census data on the most common female and male first names in the United States ([32] for female first names and [33] for male first names). We code the gender of officials according to the appearance of his or her first name in the census data. Officials with a first name listed as being either a female or a male name are coded as male if their first name is more frequently used for men than for women, and vice versa. A comparison of the resulting categories with the true gender categories for the 3,344 cases with information on gender indicates that the classification based on the first name is correct in 98.4 percent of the cases, and thus highly accurate. Based on the classification of all the officials in county legislative offices across the United States, we then compute the share of women in such offices per county. The results are presented in Fig 3, (a) for all counties in the United States, (b) exemplary for the state of New York, and (c) exemplary for the state of Texas. Counties for which no data were available are indicated as grey areas (in some counties only the initials of the first names are provided, and a gender classification based on the first names is thus not feasible). A script with the original code and data for this analysis are presented in the Supporting Information (S1 Code and S1 Data).

The mapped descriptive statistics clearly indicate that male dominated county legislative offices are more prevalent than female dominated ones. Female representation is generally stronger in the Western states compared to the ones in the Heartland and down to Texas. In the county Genesee in the State of New York, the share of women in local politics reaches over 50 percent, while in the neighboring counties clearly less than half of the local politicians are female. Based on the figures, women-led county offices are thus easy to detect.

## Replicability: Raw data access and preparation documented with pvsR

Replication is the hallmark of science. Evidence in favor or against a theory is built up when some hypothesis is confronted with the ‘same’ analysis over and over again. Thereby, a pure or statistical reproduction involves a re-estimation based on the same model and either the same data sample or at least the same underlying population. In contrast, following the taxonomy of [34], scientific replications involve similar models, but different data. The new information sources improve the efficiency of the scientific process for sampling data and performing statistical replications. With unique data sets based on big public data, it is key that research and, in particular, the data-generating process is fully reproducible. OSIs help to establish a high standard of replicability.

Indeed, the pvsR package does not only facilitate data compilation from PVS’ rich database, but it is also particularly well designed for replicable research. First, it is written in the R programming language, guaranteeing platform independence and free availability. Second, the functions in pvsR are wrapped around and named after the query methods and classes of the PVS API, which facilitates interpreting the use of these functions without actually knowing pvsR or having access to the PVS API (to see what data a certain function provides, the underlying PVS API method can simply be looked up in the official description: http://api.votesmart.org/docs/index.html. Additionally, users with access to the PVS API can verify what data is used by querying it with an alternative device, such as a web browser). Finally, all pvsR functions return the data in a format for statistical analysis.

We briefly demonstrate how pvsR can be used to replicate existing studies at low cost as well as to write a fully reproducible empirical analysis. Specifically, we refer to a study [35] that analyzes the voting behavior of so-called lawyer-legislators in tort issues at both the federal and the state level in the United States. The basic hypothesis states that due to professional and private interests, lawyers in parliament support an extended tort system that renders litigation more attractive for solving liability questions in society. We replicate the core results provided in [35] by testing the same hypothesis in a similar fashion but with a different sample. The code of the example serves as an illustration of how research with pvsR is automatically and sufficiently documented for future reproduction/replication. The analysis is performed step by step, from the compilation and cleaning of the raw data to the econometric test of the hypotheses. The following code shows the key aspects of the data gathering process. The complete code for this step is provided in the Supporting Information (S2 Code).

We begin with pvsR to search for bills that deal with the issue of liability. Based on the returned data on the bills, we obtain the respective roll-call records as well as biographical information on all representatives participating in the vote.

*> bills <- Votes.getBillsByYearState(year = 2000, stateId = “NA”);*

*> # string matching of the term “Liability” in the title column*

*> bills <- bills[grep(“Liability”, bills$title),]*

*> # get details of the bill, extract data on bill actions separately*

*> bill <- Votes.getBill(bills$billId, separate = “actions”)*

A brief inspection of the data reveals that a bill has been found that matches the criteria needed for a replication of [35] and that it has been voted on in Congress. After extracting the relevant action-ID associated with the votes, we fetch the roll-call records.

*> # get the actionId related to the passage of the bill*

*> aId <- bill[[“actions”]]$actionId[bill[[“actions”]]$stage == “Passage”]*

*> votes <- Votes.getBillActionVotes(actionId = aId)*

After removing absentees and delegates from the roll-call record, we obtain biographical data on all representatives participating in the votes:

*> bio <- CandidateBio.getBio(candidateId = votes$candidateId)*

**Fig 4:**
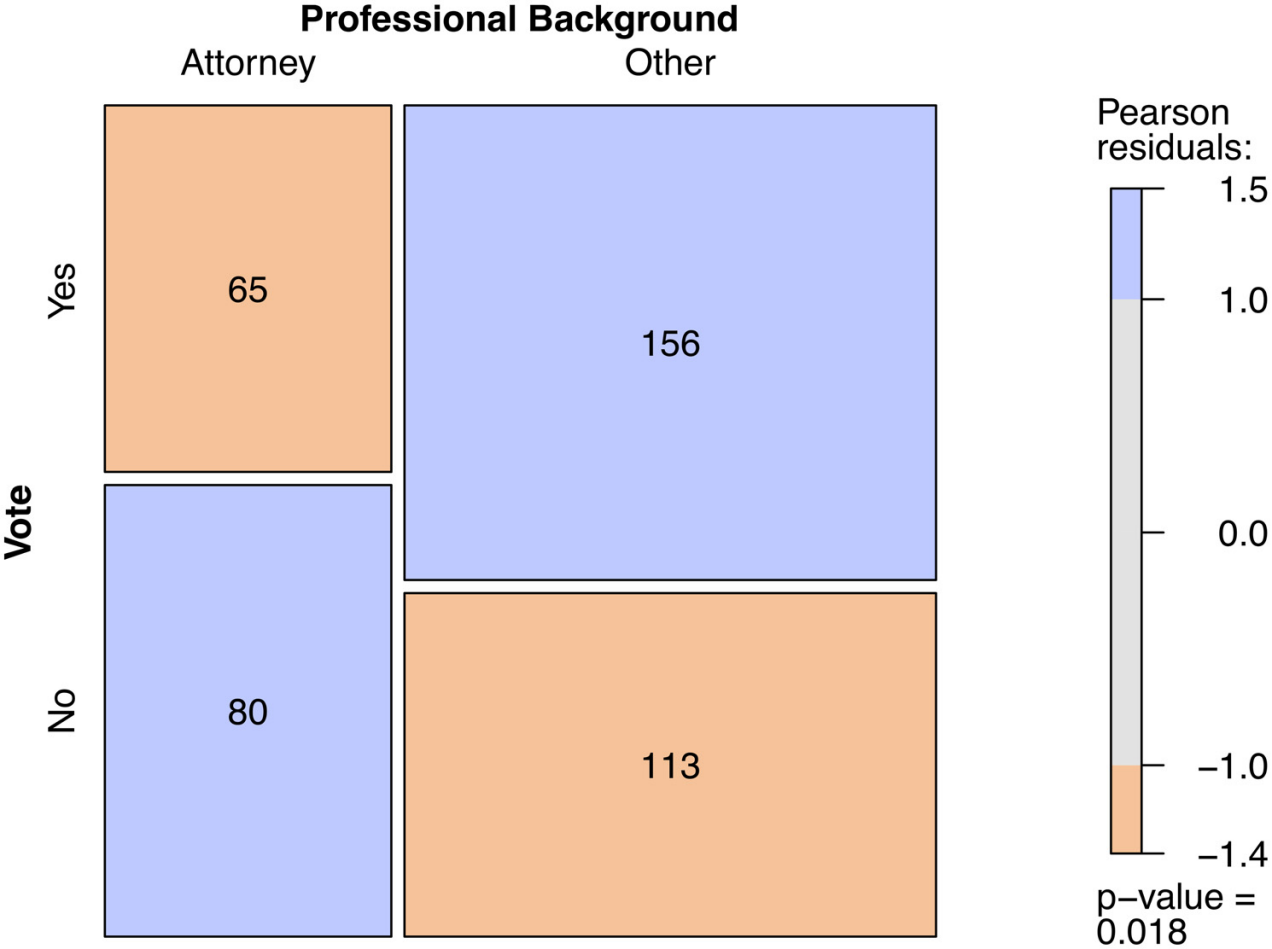
Mosaic plot of votes by professional background. *Notes:* Graphical display of the association between the professional backround of a legislator and his or her voting behavior (based on a Pearson chi-squared test of independence, p-value reported in plot). The width of the cells represents the relative share of the professional background categories, while the hight of the cells refers to the proportion of votes within each category. The shading of the cells refers to the sign and magnitude of the respective Pearson residuals. Additionally, the number of observations per cell is presented. *Data source:* Own compilation based on Project Vote Smart.

In a second step, we prepare the retrieved data for statistical analysis. We merge the data on legislators’ voting behavior with biographical data on their personal characteristics. Based on this raw data set, we create an indicator variable that is equal to 1 if a legislator voted yes, and 0 otherwise. An additional indicator variable is equal to 1 if the legislator’s candidate profile mentions a professional background as “attorney” or “lawyer”, and 0 otherwise. A third variable indicates whether a legislator is “Republican” (1) or not (0). Note that, for the sake of the example, the coding of the indicator variables is kept deliberately simple. In other applications, more sophisticated search algorithms to code the professional background of legislators might, of course, be more appropriate. A first look at the data in the form of a mosaic plot presented in Fig 4 reveals the same pattern as the one identified by [35]. Legislators with a professional background as attorneys seem to be systematically less likely to vote in favor of tort reforms that aim at restricting liability.

**Fig 5:**
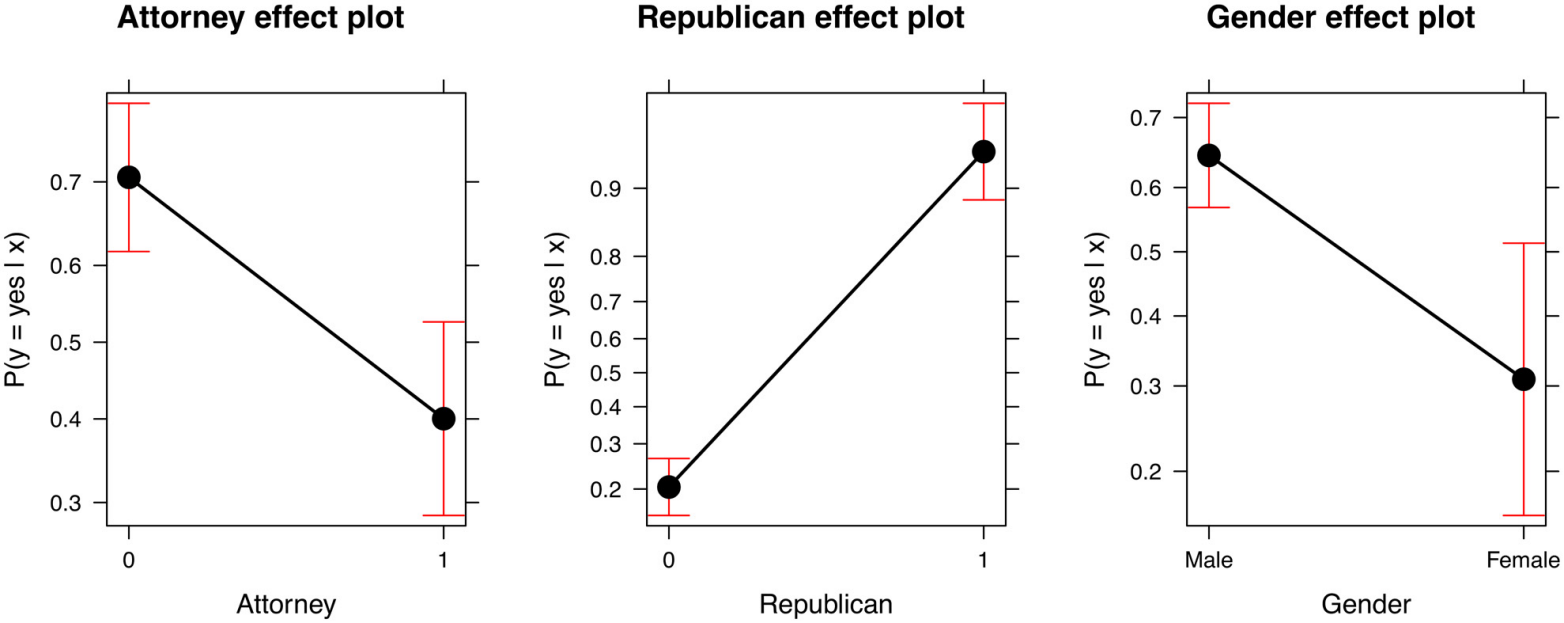
Effect plots. *Notes:* Effect plots based on the estimated logit model with specification (3) presented in Table 1. The y-axis is labelled on the probability scale. The red error bars display a 95-percent confidence interval around the estimated effect. *Data source:* Own compilation based on Project Vote Smart.

This simple analysis does, however, ignore other factors that might simultaniously influence a legislator’s voting decision and might be correlated with his or her professional background. According to [35], party affiliation and gender are potentially important predictors of voting behavior in the context of tort reform. We, thus, take these variables into consideration and refine our replication study with an estimation of the following statistical model:

$$p_{i}=P\left(y_{i}=1|x_{i}\right)=\Lambda \left(x_{i}^{\prime }\beta \right)=\frac{\exp(x_{i}^{\prime }\beta )}{1+\exp(x_{i}^{\prime }\beta )},$$ (1)

where *p_i_* is the probability that legislator *i* votes yes, *y_i_* is the dependent indicator variable describing the representative’s vote, *x_i_* is a vector with the explanatory variables “attorney”, “Republican”, and gender describing legislator *i*, and *β* is the vector of regression coefficients. We estimate our logit model of voting behavior with a common maximum-likelihood approach (unlike in [35], as no complete separation occurs in the sample of this replication study). The estimated coefficients and standard errors for three model specifications are presented in Table 1. The corresponding effects for specification (3) computed with the method suggested by [36] are displayed in Fig 5. The results of the replication study are qualitatively the same as the original results presented in [35]. Republicans are ceteris paribus more likely to vote in favor of tort reforms, whereas female representatives and lawyer-legislators are systematically less likely to vote in favor of restricting tort reforms.

**Table 1:** Voting behavior in the U.S. Congress on the tort reform bill HR 2366.

**Dependent variable: Vote in support of reform = 1**			
Coefficients	(1)	(2)	(3)
Intercept	0.322 *** (0.124)	-0.967 *** (0.177)	-0.731 *** (0.190)
Attorney	-0.530 ** (0.208)	-1.083 *** (0.324)	-1.275 *** (0.333)
Republican		3.963 *** (0.344)	3.997 *** (0.352)
Female			-1.414 *** (0.470)
N	414	414	414
BIC	577.52	345.264	340.985

*Notes:* Estimated logit models. Standard errors are in parentheses. Statistical significance: * 0.1>p>0.05, ** 0.05>p>0.01 and *** p<0.01. *Data source:* Own compilation based on Project Vote Smart.

This short replication study presents in only a few lines of code an empirical test of a politico-economic hypothesis that draws on a rich, publicly accessible data source. The complete code presented in the Supporting Information (S2 Code) is fully reproducible with respect to raw data compilation (with a clear reference to the data source—implemented and documented in pvsR), data preparation, as well as econometric analysis. The processing takes approximately 1.5 minutes on an up-to-date computer with a fast Internet connection.

## Basic architecture of pvsR

This section provides a brief overview over pvsR’s core modules. While its internal functions can be described as an API client library, the full pvsR package goes beyond this.

pvsR combines the following three basic functions. First, it facilitates the automatic sending of queries to the API (the sending of HTTP requests as well as the parsing and further processing of XML documents is facilitated by already existing open source software libraries such as [37] and [38]). Second, it parses the XML documents, cleans malformed XML, passes on error messages from the PVS API, and warns about missing and/or incomplete data. Third, it translates the received tree-structured data into either one data table (with the units of observation described in rows and the attributes describing the units in columns) or a corresponding relational database-like design with several tables (containing primary keys to emulate relationships between the tables).

All request functions for an interactive session such as Officials.getByLastname() integrate all three of these steps and are vectorized (i.e., implicitly loop over a vector of arguments to a function parameter). In addition to the usual request functions wrapped around (and vectorized over) the PVS API methods, pvsR provides several high-level functions such as getAllLocalOfficials() that facilitate the compilation of large data sets via the PVS API. These functions indicate the progress of a series of requests to the API in the R console, while the requests are split up into batches and are periodically saved on the local hard disk to keep the assignment of RAM to the pvsR task at a moderate level. Moreover, this procedure ensures that network interruptions can only do minimal harm to large requests.

**Fig 6:**
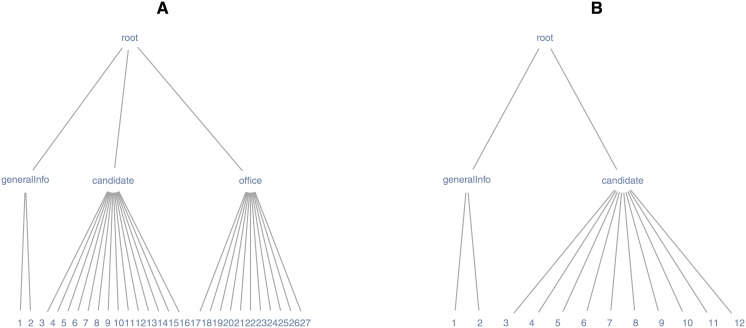
Tree structure of PVS data I. *Notes:* Reinold-Tilford tree-graph representing the document structure of data returned from two CandidateBio.getBio queries. The nodes in the graph represent XML-elements (-tags). The edges represent parent-child relationships between nodes/elements and thereby capture the nested structure of the documents. *Data source:* Own compilation based on Project Vote Smart.

**Fig 7:**
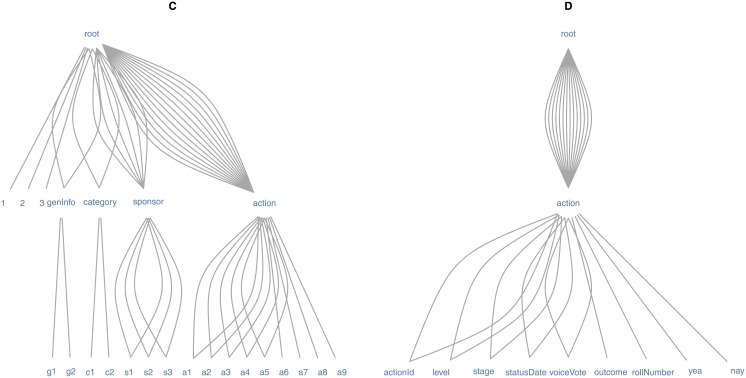
Tree structure of PVS data II. *Notes:* Reinold-Tilford tree-graph representing the document structure of data returned from a Votes.getBill query. The nodes in the graph represent XML-elements (-tags). The edges represent parent-child relationships between nodes/elements and thereby capture the nested structure of the documents. Adjacent parallel edges between the same two nodes represent the number of leaves contained in the child node. *Data source:* Own compilation based on Project Vote Smart.

The tree-structured data from the PVS API are initially not formatted for scientific analysis, and the pvsR functions are vectorized over many parameter arguments. The transformation of this data to a flat representation is thus not straightforward in every case. General methods for representing arbitrary tree-structured documents as data records with a flat structure are still the subject of current research in the field of XML data mining (see, e.g., [39] as well as the research on how to map the data in a set of XML-documents to a set of linked tables in the form of a relational data base scheme; e.g., in [40]). Exceptions in R are functions that make an attempt to directly translate XML documents with a simple structure (i.e., an XML document based on one table) to a flat data representation (see, e.g., xmlToDataFrame() in [37]). However, such functions fail in several cases or are less efficient than a specific implementation when used with PVS API data, as XML documents from web APIs can exhibit a rather complex structure. The complexity is related to the fact that XML documents from the PVS API do not necessarily represent only one underlying data table when translated to a flat representation. They might, instead, include additional metadata or nested tables. Figs 6 and 7 illustrate this point for data fetched with two pvsR functions and PVS API methods, respectively. The XML documents received from the PVS API are graphically represented in a tree-like graph, where nodes represent data elements. In the example presented here, the graph layout is based on the Reingold-Tilford algorithm (see [41] and [42]).

Each element on the lowest level in the tree (so-called “leaves”) represents one variable containing the respective data values (the original variable names in Figs 6 and 7 are partly abbreviated or replaced by digits for the sake of visualization). The root or “parent” element of all other elements represents the whole document. The edges as well as the elements between the root and leaf elements represent how the variables (leaves) are nested within the document. The number of edges between any two elements represent the number of leaves (i.e., data records) within the lower of the two elements. Graph A in Fig 6 represents an XML document returned by the CandidateBio.getBio method of the PVS API. The document describes with 27 variables, one candidate who currently holds an office. Variables 1 and 2 contain general information about the candidate’s profile, such as the URL to the candidate’s webpage on votesmart.org, variables 3 to 16 describe the candidate (e.g., his/her name and educational background), variables 17 to 27 describe the office the candidate holds. Based on the same request method, the XML document in graph B uses 12 variables to describe a candidate who does not hold an office. Thus, while the same type of request is sent to the PVS API in both cases, the returned XML documents differ significantly. However, in both cases the tree structured data can be thought of as one data table with one row and 27 or 12 columns, respectively. pvsR (in this case the function CandidateBio.getBio()) can handle hundreds of requests in one function call and rearranges the diverse XML documents with potentially different variables returned from the PVS API in one data frame (table).

Graph C in Fig 7 represents an XML document returned from a Votes.getBill query to the PVS API. The returned document contains data on only one bill. However, thinking of it as one data record described by several variables is less suitable than in the example before. The first three leaves as well as the nodes “genInfo” and “category” can be thought of as metadata describing the document (bill) as a whole. The remaining parent elements “sponsor” and “action” each describe one aspect of the bill with several variables (leaves). Both can be thought of as separate sub-trees (see graph D for the case of “action”) and hence as separate data tables containing several variables and data records. The respective function in pvsR (Votes.getBill()) can automatically process the different parts of the document separately and return the document as a list of three data frames (tables): one containing the metadata, one containing data on the bill’s sponsors, and one containing data related to “bill-actions” (e.g., votes; see the example in the previous section). With regard to the same query, pvsR can handle hundreds of requests in one function call and rearranges and aligns the three parts of the diverse XML documents simultaneously in three separate tables.

The scope of pvsR is thus different from a simple API client library that handles individual requests. Due to the specific nature of the data at hand (driven by the initial purpose of PVS API), pvsR should also be set into contrast to client library tools used to access scientific online databases. Such tools are applied in a context where data is provided by scientists to scientists, and data is already made available in ready-to-use data sets often with the explicit aim of improving data availability for systematic analysis. See, e.g., the R-package FAOSTAT [43], which is an interface to the FAOSTAT data repository (http://faostat.fao.org), or the R-package GEOquery [44], which is an interface to the NCBI Gene Expression Omnibus data repository (www.ncbi.nlm.nih.gov/gds). As outlined above, pvsR simplifies systematic data gathering and compilation for scientific use under rather different circumstances.

## Related work and advantages of pvsR for social science research vis-à-vis other PVS API client libraries

pvsR is the first (and currently only) client interface to the PVS API made for the R statistical computing environment. It is also the only software library capable of interacting with the PVS API that is specifically tailored for social science research and that explicitly follows our suggested OSI concept. pvsR is thus not reserved for users with a computer science or web programming background, but is, instead, explicitly provided for researchers who use R as their everyday statistical software package to analyze and visualize data. For example, pvsR users need no specific knowledge about XML, RESTful APIs, or API clients in general in order to productively work with the software. Neither do they need to know how to install software from an online Git repository hosting service such as GitHub. Please note that our general considerations on OSIs are not meant to be exclusive to the R statistical computing environment. Rather, we argue that the central features of OSIs (in particular with respect to replicability of research) are free availability, open-source software, a detailed and user-friendly documentation, as well as user-friendly implementation. All these features are aimed at data compilation for statistical analysis. In this section, we thus point to other valuable free and open-source software libraries built to interact with the PVS API. They are provided in other programming languages. We discuss their suitability as OSIs vis-à-vis pvsR.

At the time this paper was written, API client libraries which interact with the PVS API for the following four additional computing-environments were available: [24] provides a library for PHP, [45] provides a RESTful Java client, [46] provide a wrapper for the PVS API written in Ruby, and [47] provides a python client library (note that additional tools might be available in the future; PVS lists such contributions on http://votesmart.org/share/api). As these libraries are all written in a different language (and pvsR is the only one written in R), it is difficult to compare them with pvsR without also comparing these different languages and their main fields of application. The libraries [24, 45], and [46] are all written in languages primarily provided for and used by programmers rather than researchers. This becomes apparent when considering these packages as OSIs. The short documentation and example describing [24] show that the library is primarily designed for web programmers who wish to embed data from the PVS API into dynamic websites (see https://github.com/votesmart/php-votesmart/blob/master/example.php). A thorough API client library, intended for querying data from the PVS API via the Java programming language, which covers all PVS API classes, is provided by [45]. The package is documented with comments in the source code. In addition, helpful examples are provided that document how Java programmers could build on the library to write high-level functions that might facilitate the querying of data from PVS API for end-users. In a similar vein, the authors of [46] present a thorough API client library for Ruby programmers. However, it does not cover all current PVS API methods. The library’s documentation (provided via RubyGems, a package manager for the ruby language) focuses on the technical aspects of the program and clearly facilitates the use of the package for programming purposes. In comparison to pvsR, all three libraries primarily aim at a user group other than social scientists. Most importantly, for users without programming experience it might not be apparent how to employ these API client libraries in order to seamlessly integrate PVS data into a statistical analysis. This is, of course, in no way a criticism of these software contributions as such.

From the selection of PVS API client libraries that are available in languages other than R, the python library [47] comes closest to the suggested OSI concept. While the library’s intended audience is explicitly “developers” (see, i.e; https://github.com/sunlightlabs/python-votesmart/blob/master/setup.py), its focus is on fetching data from the PVS API in a format that can be considered convenient for research purposes. However, there is a central difference to pvsR with respect to data compilation. The provided methods do not implicitly loop over a vector of input arguments and automatically bind the returned and parsed XML data into one or several table-like objects. High-level functions which are comparable to pvsR’s getAllLocalOfficials() are also not provided. These aspects are central to our conceptualization of OSIs, as they substantially reduce the costs of data gathering for users who employ R or python as their everyday tool for statistical analysis. A more general advantage of pvsR in comparison to [47] is its thorough documentation, which includes for each of its functions a concise description of the function’s usage, arguments, returned values (including what variables are provided), hints to similar and/or related functions, and a working example. The complete (88 pages) pvsR-documentation is available as pdf on http://cran.r-project.org/web/packages/pvsR/pvsR.pdf. In addition, users can directly query specific function documentations via the R console (e.g., getAllLocalOfficials() or help(getAllLocalOfficials provides the specific documentation for the function getAllLocalOfficials()).

## Discussion

OSI programs, like pvsR, build the missing link between a non-scientific API (or web service) providing public data and the statistical analysis of this data. OSIs have a remarkable capacity to facilitate researchers’ access to big public data sources in the programmable web. Moreover, by simply referring to the archived OSI and supplying a short documentation of how it was used, the cost for replicating an entire empirical analysis can be reduced dramatically—not only for the statistical computations, but also for the retrieval, compilation and preparation of the data. In a broad sense, this implies a quantitative analysis architecture that allows for replicable research at low costs. The application of an OSI to public data in order to make research replicable takes the ideas proposed in [48] one step further. The published code implicitly provides the analysis and the data. OSIs might thus partly supersede the duplication of big data in journal archives in the cases where the raw data is compiled via an API. The current best practice of data availability policy is to store the final data sets centrally with a publisher or an association. Despite rapid growth in storage capacity, this practice is costly, particularly if the final data is itself big data. With OSIs, the datasets are built on demand, and it is sufficient that journals store the protocols to document the data generation process. The existing problem emphasized in [49] regarding the non-availability of important publications’ primary raw data could be alleviated. In sum, the proposal is thus for an empirical analysis architecture with OSIs as an integral component rather than individual data retrieval and compilation. These OSIs must be provided with some basic information so that they can be properly referenced. In particular, the following items of information should be included: author, year of publication, title of the program, a short note on the type (i.e., R package) and the version of the program, and a link to the code repository (an example could be: Ulrich Matter (2014). pvsR: An R package to interact with the Project Vote Smart API for scientific research. R package version 0.3. http://CRAN.R-project.org/package=pvsR).

In this paper, we introduced an OSI for the big database of the organization PVS for the R statistical computing environment. Other interesting data sources offering APIs that could be more easily used if OSIs were available are LegiScan (http://legiscan.com/legiscan), ProPublica’s Free the Files API (https://projects.propublica.org/free-the-files/api), and Buhl & Rasmussen’s API for European Union legislation (http://api.epdb.eu/), just to mention a few. The web platform programmableweb (http://www.programmableweb.com) offers a large directory of web APIs from all over the world. Around 290 APIs listed in programmableweb’s directory concern the public and political sphere. For many of them no suitable OSI is yet available for scientific use.

However, how will this “service-oriented science” [50] with OSIs for easier data access come about? This is an important question for the organization of scientific research, not least because OSIs might well increase the multiplier effect associated with publicly-funded research based on big public data. Research that uses this data is a challenge for funding agencies if data preparation consumes a large fraction of the budgeted funds. If programs for data retrieval and compilation tailored for social scientists are written in and for open source software, they can easily be used by other researchers to leverage their skills and capacity. Funding agencies might therefore welcome OSIs as a by-product of the funded research in order to create additional positive spillovers. Overall, we would like to offer three suggestions improving the conditions for the application of OSI packages. First, it is still necessary that researchers receive greater acknowledgement for freely providing research services that enable other researchers to replicate or extend scientific work. Public archives for service software like OSIs are an important step in that direction. Second, in the age of big public data from the programmable web, exact reproduction based on ‘unique’ novel data sets should be set into contrast to replication including raw data compilation based on OSIs. Finally, services have to be rewarded in the same currency as original research, i.e. with citations. Once these conditions are secured, the opportunities presented by new big public data from the programmable web may well spur social science research to new heights.

## Supporting Information

S1 Tutorial Tutorial on data compilation with pvsR. (PDF)

S1 Code R-Script to reproduce the studies on female representation in local U.S. politics and on religious affiliation in the main article. (TXT)

S2 Code R-Script to reproduce the replication study in the main article. (TXT)

S1 Data Dataset on women in local political offices in the United States. (ZIP)
