# Supplementary material for: pvsR: An Open Source Interface to Big Data on the American Political Sphere
Source: PLoS One. 2015 Jul 1;10(7):e0130501. doi: 10.1371/journal.pone.0130501 (PMC4488489; doi:10.1371/journal.pone.0130501)
Supplement: S1 Tutorial — (PDF) [file pone.0130501.s001.pdf]

Supporting Information to "pvsR: An Open Source Interface to Big Data on the American Political Sphere"

August 4, 2014

Ulrich Matter (University of Basel)

Alois Stutzer (University of Basel)

S1. Tutorial on data compilation with pvsR

The `pvsR` package is written in the R programming language, guaranteeing platform independence and free availability. It is completely open source (released under the GPL-2 license). The functions in `pvsR` are wrapped around and named after the query methods and classes of the PVS API. This facilitates interpreting the use of these functions without actually knowing `pvsR` or having access to the PVS API.[1]

Installing pvsR

The easiest way to install `pvsR` is to use the following command in the R-console (R version $\geq$ 3.0 needed):

```
> install.packages('pvsR')
```

Alternatively the package can also be downloaded as source code or binary from CRAN (http://CRAN.R-project.org/package=pvsR) and installed from the locally saved file.

[1] To see what data a certain function provides, the underlying PVS API method can simply be looked up in the official description: http://api.votesmart.org/docs/index.html. Additionally, users with access to the PVS API can verify what data is used by querying it with an alternative device, such as a web browser.

Getting started

Most query-functions in the pvsR package take a simple input variable and return the data describing this input variable in a data frame (a spread sheet like R-object suitable for statistical analysis). We start with a simple example to demonstrate the basic functionality: the function Officials.getByLastname() takes the last name(s) of one (or several) U.S. officials as an input variable and returns a data frame with a row for each official and columns describing her or him.

```
> library(pvsR)
> pvs.key <- "YOUR-PVS-KEY"

> ob <- Officials.getByLastname(lastName=list("Obama","Boehner"))
> print(ob[,1:6])

  candidateId firstName nickName middleName preferredName lastName
1        9490    Barack             Hussein        Barack    Obama
2       27015      John                  A.          John  Boehner
```

Note that it is mandatory to have a PVS API key in order to access any data (independent of the tool used to access the data). After installing and loading the pvsR package, make sure that your personal PVS API key is saved in a variable called pvs.key as shown in the code above. All query functions will automatically refer to this variable afterwards. See http://votesmart.org/share/api on how to register to Project Vote Smart in order to get an API-key.

Simple information retrieval and aggregation

Combining different pvsR-functions and basic R functions to retrieve and aggregate information about U.S. politics in order to answer simple empirical questions is straightforward. For example, what is the current share of female legislators in the California State Senate? In order to answer this question, we first use Officials.getByOfficeState() to get a data frame with all current Senators in the California State Senate. The parameter officeId specifies the office from which data should be provided, 9 stands for State Senate.[2]

[2]See http://api.votesmart.org/docs/semi-static.html for a complete list of available offices and their respective ID-number.

In a second step, we simply take the unique identification numbers (`candidateId`) of all these senators to query biographical information on each one of them with the function `CandidateBio.getBio()`.

```
> ca_senators <- Officials.getByOfficeState(stateId="CA", officeId=9)
> sen_bio <- CandidateBio.getBio(candidateId=ca_senators$candidateId)
> print(summary(sen_bio$candidate.gender))
```

```
  Male Female
    28     12
```

Note the high accuracy of the information aggregated in this manner. At the time this document was generated (September 2013), the total count of senators was 39. Although the California State Senate has 40 seats, 39 was absolutely correct. One of the seats (district 26) was vacant at that point in time ([?]). The relatively simple retrieval and aggregation of high-quality data on U.S. politics from the PVS API via `pvsR` has the potential to replace common survey practices in the field of political science.

Reproducible research

With a slightly extended example, we demonstrate how `pvsR` can be used to write a fully reproducible empirical analysis. The example is based on [?]; i.e., an analysis in which the voting behavior of lawyer-legislators is investigated. They empirically test whether U.S. legislators with a professional background as attorneys vote with a higher probability against tort reforms that potentially harm their business (restrict liability in tort cases) than the average legislator with a different professional background.

We begin with `pvsR` to search for bills, that deal with the issue of liability. Based on the returned data on the bills we get the respective roll call records, as well as biographical information on all representatives participating in the vote.

```
> bills <- Votes.getBillsByYearState(year=2000, stateId="NA");
> # string matching of the term "Liability" in the title column
> bills <- bills[grep("Liability", bills$title),]
> # get details of the bill, extract data on bill actions separately
> bill <- Votes.getBill(bills$billId, separate="actions")
```

```
> # verify relevance of found bill:
> print(strtrim(as.character(bill[["main"]]$officialTitle), width=82))

[1] ""

> # get the actionId related to the passage of the bill
> aId <- bill[["actions"]]$actionId[bill[["actions"]]$stage=="Passage"]
> # get the roll call records:
> votes <- Votes.getBillActionVotes(actionId=aId)
> # inspect the returned roll call record:
> head(votes,3)

  candidateId     candidateName officeParties action actionId
1       26827 Abercrombie, Neil    Democratic    Nay     8240
2       26970    Ackerman, Gary    Democratic    Nay     8240
3         441  Aderholt, Robert    Republican    Yea     8240

> summary(votes$action)

Did Not Vote           NA          Nay          Yea
          21            5          193          221

> # remove absentees and delegates:
> votes <- votes[votes$action=="Nay" | votes$action=="Yea",]
> # get biographical data on the representatives participating in the vote:
> bio <- CandidateBio.getBio(candidateId=votes$candidateId) #this step might take a minute
> # have a look at the names of some biographical variables:
> names(bio)[15:20]

[1] "candidate.gender"     "candidate.family"     "candidate.homeCity"
[4] "candidate.homeState"  "candidate.education"  "candidate.profession"
```

In a second step, we prepare the retrieved data for statistical analysis. All functions used from here on are basic R functions that are unrelated to the `pvsR` package. Note that, for the sake of the example, the coding

of the additional indicator variables is kept deliberatly simple. In other applications, more sophisticated search algorithms might, of course, be more appropriate.

```
> # combine the biographical data with the votes:
> leg_vote <- merge(votes, bio, by="candidateId")
> # code additional variables for statistical analysis:
> leg_vote$yes <- 0
> leg_vote$yes[leg_vote$action=="Yea"] <- 1
> leg_vote$attorney <- 0
> leg_vote$attorney[grep("Attorney", leg_vote$candidate.profession)]<-1
> leg_vote$attorney[grep("Lawyer", leg_vote$candidate.profession)]<-1
> leg_vote$republican <- 0
> leg_vote$republican[leg_vote$officeParties=="Republican"] <- 1
```

Finally, we fit and test an empirical model of voting behavior.

```
> model <- yes~ attorney + republican + candidate.gender
> fit <- glm(model, family=binomial(link="logit"), data=leg_vote)
> print(summary(fit)$coef)

                        Estimate Std. Error   z value     Pr(>|z|)
(Intercept)           -0.7310102  0.1895185 -3.857197 1.146945e-04
attorney              -1.2751299  0.3333074 -3.825687 1.304080e-04
republican             3.9970766  0.3518890 11.358914 6.697189e-30
candidate.genderFemale -1.4137394  0.4699506 -3.008272 2.627377e-03
```

The example above presents in only 20 lines of code an empirical test of a politico-economic hypothesis that draws on a rich, publicly accessible data source. It is fully reproducible with respect to data compilation (with a clear reference to the data source - implemented and documented in `pvsR`), data preparation as well as econometric analysis. The processing takes approximately 1.5 minutes on an up-to-date computer with a fast Internet connection.
